# Supplementary material for: Thirteen dubious ways to detect conserved structural RNAs
Source: IUBMB Life. Author manuscript; Available in PMC 2024 Jul 10. (PMC11234323; doi:10.1002/iub.2694)
Supplement: supplementary_material [file NIHMS1907110-supplement-supplementary_material.gz › supplemental_material/Table1/CRS.structural.tar.gz!/selected/selected_noRfam/noNUMTs/cmf.M0914129/MyM0914129/R-scape_cmsearch/cmf.M0914129_ext_liftover.hg38.R2R.sto.pdf]

cmf.M0914129_ext_liftover.hg38

5′-○○○○○○○○○○○○○○○○●R●●YYYCYCY●●U●●YARRGRYRYY●●CA●RR●Y●YRRRG●YYY●YR○○○○○○○○○○○○○○○○○○○○○○○○○○○○○○○○○○●R●R●G●RYCY○YRYRYAAARY●●CYYCY●YYY●●●●AYRY●CCYGUR●RUACA●●C●●●Y●CA●YRRCAYRYR●●YAY●CAGARAYR●ACAYRYGYRR●YCCAYR●CCYY●●RRYY●YGAA●YYY●●GY●YRGGG●RGYYUGRGCYRG●UYYCYUG●GCAAAYACAACUCRUGCUGUCYUCUCYARA●YCYAUUGUGAAAAYCAAACACUUGYYAG○
